# Sexual Dimorphisms of Adrenal Steroids, Sex Hormones, and Immunological Biomarkers and Possible Risk Factors for Developing Rheumatoid Arthritis

**DOI:** 10.1155/2015/929246

**Published:** 2015-11-26

**Authors:** Alfonse T. Masi, Azeem A. Rehman, Laura C. Jorgenson, Jennifer M. Smith, Jean C. Aldag

**Affiliations:** ^1^Department of Medicine, University of Illinois College of Medicine at Peoria (UICOMP), One Illini Drive, Peoria, IL 61656, USA; ^2^University of Illinois College of Medicine at Peoria, Peoria, IL 61656, USA

## Abstract

Innate immunity and immunological biomarkers are believed to be interrelated with sex hormones and other neuroendocrine factors. Sexual dimorphism mechanisms may be operating in certain rheumatic and inflammatory diseases which occur more frequently in women than men, as rheumatoid arthritis (RA). Less data have been available on altered interrelations of the combined neuroendocrine and immune (NEI) systems as risk factors for development of certain diseases. In this study, serological interrelations of NEI biomarkers are analyzed before symptomatic onset of RA (pre-RA) versus control (CN) subjects, stratified by sex. Sexual dimorphism was found in serum levels of acute serum amyloid A (ASAA), soluble interleukin-2 receptor alpha (sIL-2R*α*), and soluble tumor necrosis factor receptor 1 (sTNF-R1). Multiple steroidal and hormonal (neuroendocrine) factors also showed highly (*p* < 0.001) significant sexual dimorphism in their assayed values, but less for cortisol (*p* = 0.012), and not for 17-hydroxyprogesterone (*p* = 0.176). After stratification by sex and risk of developing RA, differential NEI correlational patterns were observed in the interplay of the NEI systems between the pre-RA and CN groups, which deserve further investigation.

## 1. Introduction

Clinical experimental [1–3] and prospective epidemiological studies [4] suggest that dysregulation of adrenocortical or gonadal function may predispose to developing rheumatoid arthritis (RA). Serum adrenal androgen (AA) steroid levels were lower in a minority of women who had premenopausal onset of disease, compared to matched control (CN) females [2]. A greater minority of women who subsequently developed rheumatoid arthritis (pre-RA cases) had combined low serum cortisol and low androstenedione concentrations than matched cohort CN females [4]. Multicompartmental (multizonal) relative adrenocortical insufficiency was suspected in a minority subset of pre-RA women, which may predispose to developing the disease [4].

The immunological network, including serum acute phase proteins (APPs), inflammatory cytokines, their receptors, and antagonists, is integrated [5, 6]. Pro-inflammatory cytokine signaling activates APPs and also influences their receptor concentrations under complex biological systems control mechanisms during health and varied clinical diseases [5, 6]. Tumor necrosis factor-alpha (TNF-*α*) and interleukin-6 (IL-6) are increased in chronic inflammatory diseases, like RA [7]. In a domestic cohort study [8], elevated serum C-reactive protein (CRP) levels (8+ mg/dL) were more frequently (*p* = 0.010) found in 8 (17.4%) of 46 pre-RA than in 9 (5.0%) of 179 CN subjects. In a separate case-control cohort study of blood donors in Netherlands [9], slightly higher median CRP concentrations were found in multiple serum samples from 79 pre-RA compared to 79 matched CN subjects. The differences were significant within a 5-year interval between serum testing and onset of clinical symptoms, but not in longer 1-year periods, extending to 15 years (median 7.5 years). Serum acute phase proteins, inflammatory cytokines, and other immunological components differ by age and sex and may be related to such host predispositions in inflammatory diseases, like RA [8]. The aim of this study was to analyze a large community cohort database for relations of age and sex to both neuroendocrine and immunologic (NEI) biomarkers, particularly as they may relate to sexual dimorphism and predisposition to clinical onset of RA.

## 2. Methods

### 2.1. The RA Precursors Study (RAPS) Neuroendocrine Immune (NEI) Database

The RA Precursors Study (RAPS) was initiated at this institution in 1991 [4, 8, 10]. Baseline data and sera were donated by* Operation CLUE I*, a community-wide prospective study. A comprehensive serum neuroendocrine panel was assayed in a university referral laboratory on a community-based cohort of pre-RA and CN females in 1992 and 1994 [4] and on a male cohort of study subjects in 1996 [10]. Female study subjects were derived from a community-based cohort of 12,381 residents of Washington Country, Maryland, enrolled in 1974 [4, 8]. A total of 36 baseline (1974) pre-RA females were identified who later developed clinical RA (3–18, median 12 years after entry). The stored (−70°C) baseline sera were anonymously coded and assayed in matched sets of one pre-RA and four CN subjects without knowledge of subject status. The sole rheumatologist in the cohort community had diagnosed and confirmed the RA cases according to American College of Rheumatology (ACR) 1987 revised classification criteria [11] and European League Against Rheumatism (EULAR) recommendations for predisease criteria [12]. A total of 144 CN women were closely matched (4 : 1) to the 36 pre-RA cases at entry to the cohort. The first batch of study sera was assayed in 1992 on 14 pre-RA and 56 CN females [4, 8, 10]. The second batch of sera was analyzed in 1994 and included the remaining 22 pre-RA and 88 CN subjects [4, 8, 10]. A serum fractionation procedure was developed specifically for this study to permit measurements of 12 steroids in duplicate from 1 mL of serum, as described [4].

The CLUE I 1974 entry cohort had enrolled 8,680 males and 12,381 females of Washington County, Maryland, USA. The RAPS database currently includes 90 males (18 pre-RA and 72 CN) and 180 females (36 pre-RA and 144 CN) study subjects, in a ratio of 1 pre-RA : 4 CN. The UICOMP Institutional Review Board approved this research for assurance of confidentiality. Clinical onsets of RA in male and female cases occurred 3 to 20 years (median 12 years) following the 1974 entry into the cohort (1977 to 1994) [8]. No matched CN subject had a diagnosis of RA in the community rheumatologist's practice. The non-RA cohort CN subjects were matched to pre-RA cases on sex and race (all Caucasians) and usually within one year of age at entry. The selected CN subjects were the closest in chronological sequence of enrollment in the cohort to the pre-RA, analogous to another case-control study [13]. Case or control subjects who had known cancer diagnoses during follow-up were excluded from the RAPS database. Their sera were reserved to study cancer biomarkers, which is the primary purpose of Operation CLUE [14–16].

### 2.2. Assay Methods for the Comprehensive Panel of Serum Steroids and Hormones

A comprehensive panel of adrenal and sex steroids was assayed in males and females, using previously developed and described methodology [4]. Intra-assay percentile coefficients of variation (% CVs) were all less than 12%, as the measurement criterion for acceptability. Too few batches of assays were performed in the 1992 or 1994 sets to analyze interassay variability among females [4]. The female sera were assayed in separate 1992 (early set) and 1994 (late set) batches. The steroid and hormonal results of the smaller number of 1992 first-set samples (*n* = 70) were normalized by their mean values to the means of the larger 1994 second-set samples (*n* = 110), after stratification on pre- and postmenopausal cohort entry status [4]. The number of assayed steroids in the female profile was larger than in males, including the majority of the non-17-hydroxylated steroids (mineralocorticoid pathway), as previously reported [4]. Accordingly, a minority of female subjects had insufficient sera to perform the full panel of the other hormonal assays completed in the males [4]. The minority of missing steroid values in females were imputed, as described below. In both sexes, assay priority was given to cortisol, dehydroepiandrosterone sulfate (DHEAS), luteinizing hormone (LH), and prolactin (PRL), which were assayed completely in both male and female subjects.

The male sera (*n* = 90) were assayed completely in 1996 [4, 8, 10] and those values were analyzed without normalization. In males, sera were sufficient for total assays of two sex hormones, total testosterone (T) and estradiol (E2), two C19 androgenic steroid precursors (DHEA and androstenedione), cortisol, two C21 17-hydroxylated glucocorticoid (GC) precursors (17-hydroxy pregnenolone and 17-hydroxy progesterone), and two pituitary hormones (LH and PRL). In females, sera were sufficient for total assays of cortisol, DHEAS, and three pituitary hormones (FSH, LH, and PRL), but not for the other steroids [10].

### 2.3. Several Reference Laboratories Performed Immune Assays from 1992 to 1996

Immune assays were performed in males and females by several reference laboratories, using developed and described methodology [8]. As funding was secured during the interval of 1992 to 1996, the study subject sera were periodically utilized for assays of acute phase proteins and cytokines in the referral laboratories. In analyses of female immune biomarker assays, the reported first-set values were normalized to the second-set results by the respective differences in their means, after stratification on pre- and postmenopausal cohort entry status. Although all female cytokine assays were performed at Specialty Laboratories, Inc. (SLI), Santa Monica, CA, in April 1995, results on first- and second-set women were determined in separate runs. For C-reactive protein (CRP) assays, both the first set performed at Northwestern University and the second set, including ASAA results, performed at Boston University [8] were adjusted to the second-set female values for combined analyses.

### 2.4. Statistical Methods

All steroidal and hormonal (neuroendocrine) values were first transformed to natural logs to improve their symmetry and distributions. Frequency distributions of all values were examined for acceptability of unimodality and symmetry features [4]. Extreme outliers were identified in several variables, particularly the acute phase proteins, which is expected for these sensitive and highly reactive tests [17]. Outliers were assigned to the respective lower and upper ranges in the frequency distributions [18]. The log-transformed NEI values were further converted to* z*-scores to standardize their variances within the 3 separate subject subsets, that is, early-set females, late-set females, and males [8], and within a merged total data set. The* z*-score values were almost always distributed ±2 or ±3 standard deviations (SDs).

Multiple imputation (MI) technique was utilized to enter a minority of acceptable values into the 3 respective data sets containing missing values at random [19, 20]. The MI technique was performed using SAS 9.2 Software (SAS Institute Inc., Cary, NC) [21]. Since the data are assumed to have an arbitrary missing data pattern, a Markov chain Monte Carlo (MCMC) method was used when conducting MI with SAS [21]. Then, 10 imputed data sets for each variable were systematically analyzed to derive a single mean value for each of the variables with missing entries, using the IBM SPSS 21.0.0.0 (IBM SPSS, 2012) program AGGREGATE [22]. Frequency distributions of the imputed values were always closely similar to the originally reported values for each variable.

Bivariate and multivariate correlational techniques were performed, including stratification of variables by sex and RA risk status. Logistic regression was used to explore relationships when the outcome was dichotomous. Multiple regression was used when the outcome was interval. Network or pathway interrelations of the NEI systems components were explored using bivariate correlational patterns of the 5 NEI and baseline age variables included in the linear regression models. The dependent and independent bivariate *β*-coefficients in regression models included age, interleukin-1*β* (IL-1*β*), interleukin-1 receptor antagonist (IL-1ra), androstenedione (adione), testosterone, and cortisol, which were used to approximate the network linkages. Age was always the independent variable in each model. In paired associations, for example, androstenedione and IL-1*β*, the *β*-coefficients and *p* values of the designated dependent variables in the models were entered in the upper panel of a composite correlational matrix. The second encounter of the respective paired correlation was entered into the lower panel. All *p* values of the respective 10 paired correlations are equal in the upper and lower panels.

Principal component analysis (PCA) was performed using oblique rotation in IBM SPSS 21.0.0.0 (IBM SPSS, 2012) program. The variable loadings on the extracted principal components permit labeling and comparison of matrix patterns among subgroups, for example, females versus males and pre-RA versus CN subjects. Strengths of loadings within components and their differences in patterns when analyzed by total subjects and by subsets of sex and RA risk were evaluated in construction of network diagrams. In this exploratory study, a significance level of *p* ≤ 0.050 was accepted without adjustment for multiple comparisons [23].

## 3. Results

### 3.1. Reported Values of Serum Inflammatory Biomarkers Assayed by Reference Laboratories

Sex differences in serum immunological and inflammatory biomarker values were found for ASAA, sIL-2R*α*, and sTNF-R1 (Table 1). The ASAA and sIL-2R*α* values were significantly (*p* < 0.010) greater in females, whereas sTNF-R1 was highly (*p* < 0.001) significantly greater in males, among the total subjects and separately in the CN and pre-RA subgroups (Table 1). The models did not include IL-6 assays, performed only in females.

### 3.2. Logistic Regression Models to Identify Independent Inflammatory Biomarkers of Sex

Logistic regression using sex as the dependent outcome variable and age as an independent variable confirmed that the preceding biomarkers independently predicted sex in the total sample when entered either singly or combined (Table 2) as well as in the smaller pre-RA subject sample (data not shown). In the CN subjects, similar results as the total sample were found, except sIL-2R*α* prediction was not quite (*p* = 0.051) significant.

### 3.3. Reported Serum Steroidal and Hormonal Values Assayed by Reference Laboratories

Highly (*p* < 0.001) significant sex differences were found in the total sample for each of the steroidal and hormonal values, except to a lesser degree for cortisol (*p* = 0.012) and not for 17-hydroxy progesterone (*p* = 0.176) (Table 3). Logistic regression was next performed using sex as the dependent outcome variable and including baseline age (Table 4). Each of the above-mentioned significant neuroendocrine values (Table 3) independently predicted sex, except for cortisol (*p* = 0.314) and PRL (*p* = 0.292). When subjects were stratified into pre-RA and CN subgroups, cortisol predicted sex in the CN (*p* = 0.025), but not in the smaller sample of pre-RA (*p* = 0.785) subjects (data not shown).

Of note, when age was included in the logistic regression model, higher 17-hydroxyprogesterone values predicted (*p* = 0.010) male sex (Table 4). In the total and control subjects, logistic regression models including age confirmed the above-mentioned sexual dimorphic differences in the individual steroidal and hormonal values. In the smaller pre-RA sample (*n* = 46), the logistic regression model did not execute with the preceding 9 variables but did provide estimates when 17-hydroxypregnenolone, cortisol, LH, and entry age were excluded. That model yielded significant prediction of the sex outcome for DHEA (*p* = 0.049), 17-hydroxyprogesterone (*p* = 0.049), and DHEAS (*p* = 0.004) and nearly (*p* = 0.053) significant prediction for androstenedione.

### 3.4. Steroidal and Hormonal Variables Predicting Sexually Dimorphic Inflammatory Biomarkers

To determine if any of the 5 neuroendocrine variables might independently predict levels of the 3 sexually dimorphic serum inflammatory biomarkers (Table 2), each of the latter inflammatory biomarkers was included as dependent variable in multiple linear regression models (Table 5). As may be expected for sIL-2R*α*, which had the least significant (*p* = 0.045) sexual dimorphism, it was predicted only by DHEA in the total subjects (*p* = 0.044) and by 17-hydroxypregnenolone in pre-RA subjects (*p* = 0.015). Both ASAA (*p* = 0.010) and sTNF-R1 (*p* < 0.001) are strongly dimorphic and both were more frequently predicted by 17-hydroxypregnenolone and androstenedione in the subset groups (Table 5). The strongest predictor of inflammatory biomarkers was 17-hydroxypregnenolone with sTNF-R1 in total (*p* < 0.001), control (*p* = 0.001), and pre-RA (*p* = 0.030) subjects (Table 5). The next strongest predictor was androstenedione with highly (*p* < 0.001) significant negative relations to sTNF-R1 in total subjects and controls and with a negative relation to ASAA outcome in pre-RA subjects (*p* = 0.015). Serum DHEA levels correlated positively with ASAA in total (*p* = 0.013) and pre-RA (*p* = 0.015) subjects and positively with IL-1ra in total subjects (*p* = 0.044). Serum DHEAS was positively correlated with sTNF-R1 in total (*p* = 0.021) and control (*p* = 0.007) subjects (Table 5).

Comparison of the pre-RA versus CN regression models revealed a significant (*p* = 0.015) negative beta coefficient of androstenedione with ASAA levels in the pre-RA cases (*B* = −0.618), which was significantly (*p* < 0.001) different from the control (*B* = 0.170, *p* = 0.209) subjects. A similar contrary sign result was observed for the negative correlation of 17-hydroxypregnenolone with sIL-2R*α* in the pre-RA (*B* = −0.595, *p* = 0.015), which differed significantly (*p* = 0.001) from the CN (*B* = 0.021, *p* = 0.862) subjects. No other significant differences were observed between the study groups.

### 3.5. Steroidal and Hormonal Variables Predicting Inflammatory Biomarkers Independently from Serum E2/T Ratio and Subject Sex Status

The ratio of serum estradiol to testosterone (E2/T) levels and subject sex status were further included in the linear regression models (Table 6) to probe if those indicators of sex may independently predict levels of the inflammatory biomarkers or displace preceding neuroendocrine predictors of sexually dimorphic inflammatory biomarkers observed in Table 5. The E2/T ratio and the sex variable were significant independent predictors of ASAA outcome in the total subjects and eliminated the previous significant predictors of 17-hydroxypregnenolone (*p* = 0.023) and DHEA (*p* = 0.013). The sex indicators did not significantly predict sIL-2R*α* levels but eliminated the previous weak prediction by DHEA (*p* = 0.044); now *p* = 0.463 (Table 6). The sex variable, but not serum E2/T ratio, is a highly (*p* < 0.001) significant independent predictor of sTNF-R1 levels in the total subjects (Table 6). It eliminated the previous highly (*p* < 0.001) significant prediction by 17-hydroxypregnenolone (Table 5); now *p* = 0.327. However, inclusion of those two sex markers uncovered a significant independent prediction by DHEA (*p* = 0.015), which was previously not significant (*p* = 0.872), indicating a profound complexity of the interrelations. Serum androstenedione remained a significant predictor of sTNF-R1 in the total (*p* = 0.002) and control (*p* = 0.004) subjects. In the smaller pre-RA subject sample, no variable was an independent predictor of sTNF-R1 levels after addition of the sex markers (Table 6). The persistent significant independent prediction of sTNF-R1 outcome by DHEA and androstenedione in total and control subjects after addition of the E2/T and sex variables suggest prominent neuroendocrine immune (NEI) interactive mechanisms (Table 6).

### 3.6. Linear Regression of Selected Standardized NEI Systems Biomarkers (Table 7)

In this analysis, the selected immunologic variables were IL-1*β* and its receptor antagonist (IL-1ra), since a physiological alteration had been found before the premenopausal onset of RA [24]. The selected steroids, androstenedione, testosterone, and cortisol, had differed between pre-RA and matched control subjects in their associations with IL-1*β* [25]. In the regression models, age is always an independent variable, but the 5 NEI variables (IL-1*β*, IL-1ra, androstenedione, testosterone, and cortisol) were analyzed as either dependent or independent factors in bivariate correlations within the regression models (Table 7). Variances (*R* ^2^) explained (VE) in the regression models differed according to the dependent biomarker, being minimal for cortisol in females (0.063 for pre-RA, 0.075 for CN), but moderate for cortisol in males (0.451 for pre-RA, 0.411 for CN). The difference in variances explained reflects stronger cortisol interrelations in males than females. Greater variance (*R* ^2^) was explained for IL-1*β* in pre-RA versus CN (0.535 versus 0.031, resp.) and for IL-1ra (0.445 versus 0.027, resp.) (Table 7), reflecting stronger interrelations of those immune biomarkers in pre-RA versus CN subjects.

### 3.7. Approximated Network Patterns of NEI Variables in Females and Males (Figures 1(a) and 1(b))

**Figure 1:**
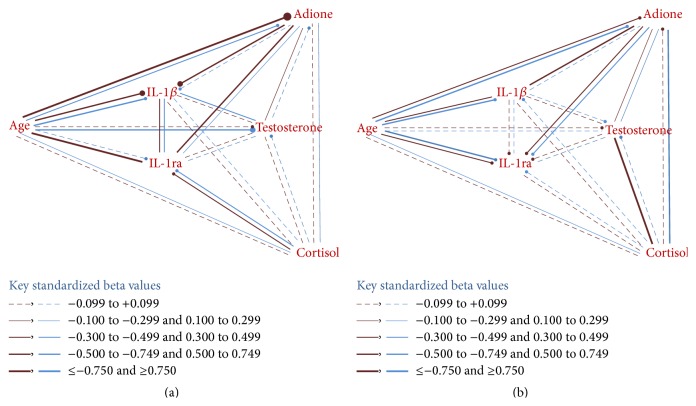
(a) Female pathway correlations showing stronger associations of age and androstenedione with the immunologic markers in pre-RA versus CN, but greater negative association of age with testosterone in CN subjects. (b) Male pathway correlations showing stronger associations of cortisol with testosterone in pre-RA versus CN, but greater association of cortisol with androstenedione in CN subjects. (a) Females: RA versus CN (pre-RA: top or left red, CN: bottom or right blue). (b) Males: RA versus CN (pre-RA: top or left red, CN: bottom or right blue).

The standardized beta (*B*) coefficients from the linear regression models of the 5 NEI variables and age (Table 7) were used to construct approximated network linkages of pre-RA and CN subjects in females (Figure 1(a)) and males (Figure 1(b)). Weights of the lines of bivariate linkages in the networks were derived from the respective beta coefficients in the linear regression models, and negative correlations are indicated by a terminal “stop sign” circle (—●). Female pre-RA have stronger interrelations of age with androstenedione, IL-1*β*, and IL-1ra than CN subjects (Figure 1(a)). Male subjects show the strongest linkages of cortisol with testosterone in pre-RA and of cortisol with androstenedione in CN subjects (Figure 1(b)). In contrast, female pre-RA and CN subjects show weakest linkages with cortisol (Figure 1(a)), consistent with their low variances explained in the respective linear regression models (Table 7). Approximated overviews of network patterns can be similarly derived for all female versus male subjects and for all combined pre-RA versus CN subjects (figures not shown). The above-mentioned stronger female interrelations of age, as indicated in Table 7 and Figure 1(a), are visually more evident in the pre-RA versus CN subject networks (figure not shown).

### 3.8. Principal Components Extraction of the Integrated Neuroendocrine and Immune Variables

The NEI variables which demonstrated sexual dimorphism (Table 6) or those which showed differential patterns of bivariate correlations between pre-RA and CN subjects (Figures 1(a) and 1(b)) were further analyzed in exploratory factor analysis methods [26]. Principal component analysis (PCA) pattern matrices were derived using oblique rotation to determine variable loadings on the extracted factors (components) of comparative subgroups, for example, pre-RA versus CN subjects (Tables 8(a) and 8(b)). This data reduction method can reveal latent relations among the variables. The imputed* z*-score database standardized to early- and late-set females and to males was used for the PCA. The pattern matrix of 214 CN subjects (Table 8(a)) has 4 principal components, which can be confidently labelled. The 1st component includes heavy loadings of the androgenic anabolic (AA) steroids, which are known to be inversely related to age. The 2nd component has heavy loadings of the sexual dimorphic cytokine receptors. The 3rd component includes heavy loadings of IL-1*β* and its receptor antagonist (IL-1ra), as an expected relation. The 4th component of CN subjects includes only cortisol and yields total variance explained (VE) of 72.7% (Table 8(a)).

In contrast, the pattern matrix of pre-RA subjects had only 3 components extracted. The 1st includes significant loadings of the AA steroids, as noted in CN subjects, but also includes IL-1*β* and cortisol. As found in the CN subjects, the 2nd component has heavy loading of the sexually dimorphic cytokine receptors. However, the 3rd component of pre-RA subjects includes loading of only IL-1ra, since the IL-1*β* physiological counterpart was extracted in the 1st component, along with cortisol. The differential pattern matrices of CN (Table 8(a)) and pre-RA (Table 8(b)) subjects suggest alterations in the underlying interrelations of the IL-1 system biomarkers and of cortisol in the pre-RA cases (Tables 8(a) and 8(b)).

## 4. Discussion

Causation mechanisms in rheumatoid arthritis are profoundly complex involving multiple genetic, age-related, sex, environmental, microvascular, neuroendocrine, and particularly immune or inflammatory pathways [27–30]. Sex hormones and sexual dimorphism control various body functions and may have predisposing or protective influences on physiological pathways or pathological processes. Dysregulations of the neuroendocrine and immune or inflammatory (NEI) systems are suspected to play a role in the development or progression of RA, in association with responses of the microvascular system [31, 32]. Differences are reported in blood levels or frequencies of suspected causal factors between affected patients and comparison subjects and differences have been observed even before the clinical onset of symptoms (pre-RA) [24]. However, such reported alterations were usually found in only a particular pathway or systemic category [24]. Analyses of cross talk between age, sex-related factors, hormones, and immune or inflammatory serum biomarkers have not previously been reported in pre-RA cases and CN subjects, to our knowledge.

In the current multivariable analyses, examples of significant sexual dimorphisms were identified in components of the NEI systems (Table 6). Adrenal androgens (DHEA and androstenedione) significantly interacted with the sexually dimorphic cytokine receptors (sIL-2R*α* and sTNF-R1), both in pre-RA in and CN subjects. Such cross talk may likely reflect normal underlying physiological control mechanisms in the NEI systems. In contrast, interactions of other NEI systems components differed significantly between the pre-RA and CN subjects. In PCA, IL-1*β* and cortisol strongly loaded with the AA steroids in the 1st component by only pre-RA, not CN, subjects (Tables 8(a) and 8(b), Figures 1(a) and 1(b)). This new finding may have been contributed by previously reported lower androstenedione and cortisol levels in female pre-RA versus CN women [4]. In previous bivariate analyses [33], combined low serum cortisol (<140 nmol/L) and low testosterone (<10 nmol/L) levels were an independent risk marker for clinical onset of RA in males. In this current multivariate analysis, the association of cortisol with testosterone was stronger in male pre-RA versus CN subjects (Table 7, Figure 1(b)).

In other bivariate analyses [34], basal steroidogenesis was considered normal in pre-RA postmenopausal women and men compared to CN subjects. That result conforms generally to the current multivariate analysis; that is, the age-related AA steroids were extracted as the 1st component in PCA of both CN (Table 8(a)) and pre-RA (Table 8(b)) subjects. Loadings of the individual AA compounds were also similar. A matrix pattern difference was the additional extraction of cortisol in the 1st component only in the pre-RA cases, although it is not age-related, as are the AA steroids. The pre-RA findings in PCA may be consistent with the above-mentioned association of lower androstenedione and cortisol levels observed in pre-RA women [4].

Lower serum levels of adrenal androgens (AA) were reported in premenopausal women with RA not treated with glucocorticoids [1, 2], which also correlated with lower serum cortisol levels [4]. Early menopause was reported to be an independent predictor of RA [3]. Intrinsically low or insufficient adrenocortical and ovarian function was proposed in a minority of premenopausal onset RA patients [4], based upon the preceding findings [1–3]. In contrast, postmenopausal women revealed normal steroidogenesis before clinical onset of RA [34]. One may hypothesize that a polymorphism in adrenal and gonadal function occurs in women and may be related to predisposition and onset age of RA. Limited data are available on such intrinsic hormonal functions before or after RA onset. Further investigation is needed to understand neuroendocrine and immune (NEI) relations to RA.

The current multivariable NEI systems analyses revealed sexual dimorphism in the interaction of IL-1*β* with its receptor antagonist (IL-1ra). A significantly stronger correlation was found in females than in males as well as in CN than in pre-RA subjects (Table 7, Figures 1(a) and 1(b)). In a previous bivariate analysis [35], perturbations in serum levels of interleukin-1 beta (IL-1*β*) and IL-1 receptor antagonist (IL-1ra) were found before onset of clinical rheumatoid arthritis (pre-RA) in women with younger onset ages. Eight of the 10 sera tested from younger pre-RA women were positive for IL-1 system perturbation versus 3 (9.7%) of 31 CN (*p* < 0.001, Fischer's exact test). Perturbation of the IL-1 system was defined as either an elevated serum IL-1*β* level (1.2+ pg/mL)* without* elevation of IL-1ra or a definitely low serum IL-1ra level (<300 pg/mL). In the complex interactions of many NEI factors, as noted in the IL-1 system, the current multivariate analyses help to focus on particular linkages and to estimate comparative strengths of the various correlations.

The observed differences in multivariate networks and PCA pattern matrices of pre-RA versus CN subjects will require further confirmatory statistical analyses [26] as well as independent controlled investigation. However, bivariate correlational findings in scatterplots support the above findings. An impressive gradient of regressions of IL-1*β* (*x*-axis) and IL-1ra (*y*-axis) is found among the sex and subject groups: (1) female CN (*r* = 0.354, *n* = 144, and *p* < 0.001); (2) female pre-RA (*r* = 0.196, *n* = 36, and *p* > 0.100); (3) male CN (−0.016, *n* = 72, and *p* > 0.100), and (4) male pre-RA (*r* = −0.477,  *n* = 18, and *p* < 0.050). The difference in bivariate correlations between the combined sex CN (*r* = 0.354, *n* = 214) and pre-RA (*r* = 0.002, *n* = 54) subjects is significant (*p* = 0.017) as is in the correlations between female (*r* = 0.473, *n* = 180) and male (*r* = −0.095, *n* = 90) subjects (*p* < 0.001).

Results of current network analyses must be qualified, as their linkages are beta coefficient approximations from linear regression models, rather than from formal pathway structured equations [36], which incorporate structured differential equations and defined directions of the factor relations. Whether or not sex or the serum E2/T ratio is included in the regression models or in the PCA, the sexually dimorphic cytokine receptors (sIL-2R*α* and sTNF-R1) revealed significant independent correlations with other NEI factors (Tables 5 and 6). Those biomarkers showed similar interactions in the pre-RA and CN groups. Considering the differences found between pre-RA and CN subjects, the interleukin-1 system may be of greater interest as a predisposing risk factor to RA or as an example of physiological dysregulation in NEI biomarkers. In females, those differences may be related to androstenedione (Figures 1(a) and 1(b)). In males, enhanced correlations of cortisol with testosterone in the pre-RA subjects (Figure 1(a)) and of cortisol with androstenedione in the control group (Figure 1(b)) may reflect unrecognized control mechanisms and deserve further investigation.

Since the earlier incorporation of multivariate analytic techniques in an integrated overview of neuroendocrine factors in relation to RA risk, 10 years ago [24], the need for such methodological approach is now intensified to gain penetrating knowledge. Univariate and bivariate analytic techniques can identify differences in systems, but the complexity of multiple interactive NEI factors requires more advanced data reduction and differential methods [36]. The current effort is a preliminary probe of interplay of NEI factors which may contribute to risk of developing RA and the significant differences need to be confirmed in further penetrating analytical investigation.

## 5. Conclusion

Current multivariate analyses identified 3 sexually dimorphic inflammatory biomarkers: ASAA; sIL-2R*α*; and sTNF-R1, which correlated with the detected sexually dimorphic steroids. Significant associations of sTNF-R1 with androstenedione were observed in total and control subjects, with or without inclusion of the sex category or the ratio of serum E2/T in the regression models. Principal component analysis showed differential loadings of IL-1*β* and cortisol in the 1st component of the matrix patterns of pre-RA versus CN subjects. After stratification by sex and risk of developing RA, other differences in the interplay of components in the NEI systems were observed, which deserve further investigation.

## Figures and Tables

**Table 1:** Reported values of inflammatory biomarkers assayed by reference laboratories.

Inflammatory mediators and statistical values	Controls		Pre-RA cases		All subjects	
	Females	Males	Females	Males	Females	Males
Mean ages ± SEs (*n*)	43.9 ± 1.0 (144)	41.8 ± 1.1 (72)	43.8 ± 2.0 (36)	41.6 ± 2.1 (18)	43.9 ± 0.9 (180)	41.8 ± 0.9 (90)
C-reactive protein (CRP)						
Mean (mg/L) ± SE (*n*)	2.8 ± 0.42 (111)	2.6 ± 0.36 (68)	5.2 ± 1.92 (29)	7.3 ± 2.9 (17)	3.7 ± 1.0 (140)	3.5 ± 0.7 (85)
Median; IQR	1.8; 1.1–2.7	1.9; 0.8–2.9	1.7; 0.9–3.4	2.6; 0.7–7.9	1.0; 1.0–2.0	1.9; 0.8–3.4
Acute serum amyloid A (ASAA)						
Mean (mg/L) ± SE (*n*)	19.9 ± 8.0 (56)	3.9 ± 0.7 (68)^*∗*^	8.4 ± 1.5 (17)	4.4 ± 1.3 (17)^*∗*^	17.2 ± 6.2 (73)	4.0 ± 0.6 (85)^*∗*^
Median; IQR	6.7; 4.0–12.2	2.5; 1.6–3.8	7.5; 3.4–11.6	3.5; 1.6–4.9	6.9; 3.7–11.6	2.5; 1.6–3.9
Interleukin-6 (IL-6)						
Mean (pg/mL) ± SE (*n*)	2.6 ± 0.3 (82)	Not measured	3.5 ± 1.1 (25)	Not measured	2.8 ± 0.3 (107)	Not measured
Median; IQR	2.0; 1.3–3.1		1.6; 1.0–3.7		1.9, 1.3–3.2	
Interleukin-I*β* (IL-1*β*)						
Mean (pg/mL) ± SE (*n*)	0.9 ± 0.2 (82)	0.7 ± 0.3 (64)	0.6 ± 0.1 (25)	0.8 ± 0.5; (16)	0.7 ± 0.2 (107)	0.7 ± 0.2 (80)
Median; IQR	0.5; 0.1–1.0	0.0; 0.0–1.0	0.5; 0.2–0.7	0.5; 0.0–1.0	0.3; 0.0–0.7	0.0; 0.0–1.0
Interleukin-1 receptor antagonist						
Mean (pg/mL) ± SE (*n*)	659 ± 50 (76)	680 ± 102 (52)	553 ± 53 (25)	853 ± 180 (11)	610 ± 40 (101)	710 ± 90 (63)
Median; IQR	516; 373–831	427; 242–917	571; 331–742	723; 441–828	503; 339–751	502; 258–902
Tumor necrosis factor-*α* (TNF-*α*)						
Mean (pg/mL) ± SE (*n*)	2.9 ± 0.3 (82)	2.2 ± 0.3 (60)	3.9 ± 0.8 (25)	2.1 ± 0.2 (15)	2.3 ± 0.3 (107)	2.2 ± 0.2 (75)
Median; IQR	2.2; 1.3–3.6	1.9; 1.2–2.5	2.2; 1.4–4.9	2.2; 1.4–2.9	1.4, 0.8–2.3	1.9, 1.3–2.5
Soluble interleukin-2R*α* (sIL-2R*α*)						
Mean (pg/mL) ± SE (*n*)	1150 ± 49 (79)	905 ± 64 (58)^†^	1222 ± 71 (24)	793 ± 84 (14)^†^	1081 ± 42 (103)	883 ± 54 (72)^†^
Median; IQR	1076; 853–1313	825; 613–1022	1213; 993–1445	844; 596–966	1040; 779–1259	825; 601–993
Soluble tumor necrosis receptor I						
Mean (pg/mL) ± SE (*n*)	1194 ± 46 (79)	1715 ± 51 (60)^‡^	1169 ± 61 (25)	1708 ± 102 (15)^‡^	1031 ± 42 (104)	1713 ± 45 (75)^‡^
Median; IQR	1109; 926–1310	1618; 1483–1883	1174; 1015–1322	1873; 1444–1988	921, 727–1236	1627; 1469–1905

^*∗*^ *p* < 0.010 (ASAA, F versus M in each group); ^†^ *p* < 0.010 (sIL-2R*α*, F versus M in each group); and ^‡^ *p* < 0.001 (sTNF-R1, F versus M in each group).

**Table 2:** Logistic regression model of sex outcome, including age and serum inflammatory biomarkers, but not IL-6 assayed only in females.

Variables in the model	Total (*N* = 270)		Controls (*N* = 216)	
	Wald^*∗*^	*p* values	Wald^*∗*^	*p* values
C-reactive protein (CRP)	1.565	0.211	0.844	0.358
Acute serum amyloid A (ASAA)	11.163	0.001	9.16	0.002
Interleukin-1*β* (IL-1*β*)	0.092	0.762	0.518	0.472
Interleukin-1 receptor antagonist	0.945	0.331	1.562	0.211
Tumor necrosis factor-*α* (TNF-*α*)	0.182	0.669	1.437	0.231
Soluble interleukin-2R*α* (sIL-2R*α*)	4.032	0.045	3.813	0.051
Soluble tumor necrosis receptor 1	14.409	<0.001	10.721	0.001
Age at entry	4.037	0.045	1.924	0.165
Constant	10.724	0.001	8.257	0.004

^*∗*^1 df for each variable entered in block.

**Table 3:** Steroidal and hormonal values reported by referral laboratory and imputed to full sample sizes.

Steroid and hormonal statistical values	Controls (*N* = 216)		Pre-RA cases (*N* = 54)		All subjects (*N* = 270)^*∗*^	
	Females	Males	Females	Males	Females	Males
	(*n* = 144)	(*n* = 72)	(*n* = 36)	(*n* = 18)	(*n* = 180)	(*n* = 90)
17-OH pregnenolone						
Mean (nmol/L) ± SE	6.8 ± 0.3	11.9 ± 0.8	6.8 ± 0.6	11.8 ± 1.1	6.8 ± 0.3	11.9 ± 0.7
Median; IQR	5.7; 4.4–7.9	10.2; 7.3–15.2	5.7; 4.2–8.8	11.0; 8.7–14.6	5.7; 4.4–8.2	10.2; 7.7–15.0
Dehydroepiandrosterone (DHEA)						
Mean (nmol/L) ± SE	18.6 ± 1.0	8.5 ± 0.6	17.9 ± 1.9	7.6 ± 1.0	18.5 ± 0.9	8.4 ± 0.5
Median; IQR	15.9; 12.5–20.8	7.2; 4.8–10.3	14.8; 11.5–19.2	7.7; 3.9–9.7	15.6; 12.4–19.7	7.2; 4.7–10.1
17-OH progesterone						
Mean (nmol/L) ± SE	4.3 ± 0.3	5.0 ± 0.3	4.3 ± 0.5	5.0 ± 0.5	4.3 ± 0.3	5.0 ± 0.3
Median; IQR	3.0; 1.9–5.7	4.7; 3.1–6.3	3.6; 1.7–6.1	4.5; 3.5–6.0	3.1; 1.9–5.9	4.7; 3.2–6.2
Androstenedione						
Mean (nmol/L) ± SE	7.6 ± 0.2	2.2 ± 0.1	6.7 ± 0.4	2.2 ± 0.2	7.5 ± 0.2	2.2 ± 0.1
Median; IQR	7.2; 6.2–8.8	2.2; 1.5–2.9	6.6; 5.3–8.3	2.2; 1.4–3.0	7.1; 6.0–8.6	2.2; 1.5–2.9
Testosterone (T)						
Mean (nmol/L) ± SE	2.5 ± 0.1	18.3 ± 0.8	2.4 ± 0.2	19.2 ± 1.8	2.5 ± 0.1	18.5 ± 0.8
Median; IQR	2.3; 1.8–2.9	17.4; 13.7–23.3	2.3; 1.8–2.9	17.8; 13.3–24.3	2.3; 1.8–2.9	17.8; 13.6–23.5
Estradiol (E2)						
Mean (pmol/L) ± SE	229.5 ± 18.2	65.4 ± 2.9	251 ± 60.7	69.7 ± 6.0	233.0 ± 19.7	66.2 ± 2.6
Median; IQR	176; 80–278	66.0; 48.0–80.3	168; 83–276	68; 49.0–84.3	173; 81–278	66.0; 48.0–81.0
E2/T ratio (×10^3^)						
Mean ± SE	123.0 ± 16.5	4.1 ± 0.3	134.0 ± 36.1	4.1 ± 0.5	125.2 ± 15.0	4.1 ± 0.2
Median; IQR	80.2; 37.6–138	3.4; 2.5–5.0	76.5; 27.7–151	4.0; 2.7–4.9	78.8; 35.4–138	3.5; 2.6–5.0
DHEA sulfate (DHEAS)						
Mean (*μ*mol/L) ± SE	3.0 ± 0.2	7.3 ± 0.4	2.5 ± 0.3	7.5 ± 1.1	2.9 ± 0.1	7.3 ± 0.4
Median; IQR	2.6; 1.6–3.9	6.8; 4.8–8.8	2.3; 1.5–3.5	6.2; 3.8–11.0	2.5; 1.6–3.8	6.8; 4.6–8.9
Cortisol						
Mean (nmol/L) ± SE	233.4 ± 11.8	291.1 ± 18.7	245.2 ± 24.6	272.8 ± 35.4	236.8 ± 10.8	285.5 ± 16.7
Median; IQR	205; 155–285	255; 186–382	241; 143–329	257; 161–381	216; 155–286	255; 184–369
Luteinizing hormone (LH)						
Mean (IU/L) ± SE	23.4 ± 1.8	5.9 ± 0.3	23.5 ± 3.4	5.8 ± 0.7	23.4 ± 1.6	5.9 ± 0.3
Median; IQR	15.7; 5.4–39.5	5.1; 4.0–6.9	19.6; 4.3–36.2	5.5; 4.0–7.2	17.1; 5.3–37.9	5.1; 4.0–7.0
Prolactin (PRL)						
Mean (*μ*g/L) ± SE	11.9 ± 0.5	7.0 ± 0.4	11.0 ± 0.8	8.0 ± 1.0	11.7 ± 0.4	7.2 ± 0.4
Median; IQR	10.4; 8.0–13.4	6.1; 4.6–9.0	9.5; 8.1–13.3	7.6; 4.0–11.4	10.1; 8.1–13.4	6.3; 4.6–9.7

^*∗*^Significant (*p* < 0.001) differences in values between total females and males, except for cortisol (*p* = 0.012) and 17-hydroxyprogesterone (*p* = 0.176).

**Table 4:** Logistic regression model of sex outcome including steroidal and hormonal variables, but not testosterone and estradiol levels^*∗*^.

Variables in the model	Total (*N* = 270)		Controls (*N* = 216)	
	Wald^†^	*p* values	Wald^†^	*p* values
17-OH pregnenolone (17-OH P5)	6.686	0.010	2.586	0.108
Dehydroepiandrosterone (DHEA)	6.572	0.010	2.305	0.129
17-OH progesterone (17-OH P4)	0.899	0.343	0.666	0.415
Androstenedione	6.600	0.010	5.169	0.023
DHEA sulfate (DHEAS)	12.708	<0.001	9.596	0.002
Cortisol	1.012	0.314	5.045	0.025
Luteinizing hormone (LH)	7.076	0.008	3.576	0.059
Prolactin (PRL)	1.110	0.292	2.305	0.129
Age at entry	2.338	0.126	1.098	0.295
Constant	0.016	0.899	0.498	0.480

^*∗*^Logistic regression models of dependent sex outcome did not execute when either testosterone or estradiol values were entered.

^†^1 df for each variable entered in block.

**Table 5:** Multiple regression models of serum inflammatory biomarker outcomes including steroidal and hormonal variables that had predicted sex.

Variables in the model of total subjects	ASAA outcome		sIL-2R*α* outcome		sTNF-R1 outcome	
	(*N* = 142)		(*N* = 159)		(*N* = 159)	
	Beta^*∗*^	*p* values	Beta^*∗*^	*p* values	Beta^*∗*^	*p* values
17-OH P5	−0.216	0.023	−0.116	0.268	0.334	<0.001
DHEA	0.286	0.013	0.200	0.044	−0.013	0.872
Adione	0.022	0.853	−0.032	0.732	−0.387	<0.001
DHEAS	−0.147	0.129	−0.147	0.130	0.177	0.021
LH	0.084	0.365	0.030	0.772	−0.094	0.255
Entry age	0.226	0.016	−0.102	0.292	0.006	0.936

Variables in the model of control subjects	ASAA outcome		sIL-2R*α* outcome		sTNF-R1 outcome	
	(*n* = 113)		(*n* = 126)		(*n* = 126)	
	Beta^*∗*^	*p* values	Beta^*∗*^	*p* values	Beta^*∗*^	*p* values

17-OH P5	−0.257	0.017	0.021	0.862	0.303	0.001
DHEA	0.198	0.146	0.204	0.073	0.046	0.604
Adione	0.170	0.209	−0.092	0.390	−0.450	<0.001
DHEAS	−0.185	0.092	−0.193	0.096	0.241	0.007
LH	0.054	0.613	0.088	0.470	−0.037	0.703
Entry age	0.199	0.054	−0.054	0.631	0.038	0.668

Variables in the model of pre-RA subjects	ASAA outcome		sIL-2R*α* outcome		sTNF-R1 outcome	
	(*n* = 29)		(*n* = 33)		(*n* = 33)	
	Beta	*p* values	Beta^*∗*^	*p* values	Beta^*∗*^	*p* values

17-OH P5	0.127	0.533	−0.595	0.015	0.479	0.030
DHEA	0.496	0.015	0.367	0.094	−0.193	0.313
Adione	−0.618	0.015	0.123	0.552	−0.198	0.272
DHEAS	−0.166	0.417	0.031	0.873	0.022	0.896
LH	0.155	0.364	−0.237	0.254	−0.231	0.208
Entry age	0.210	0.307	−0.130	0.537	−0.077	0.671

^*∗*^Standardized beta coefficients from linear regression of log-transformed, reported NEI biomarkers.

**Table 6:** Multiple regression model of serum inflammatory biomarker outcomes including variables that predicted sex plus E2/T ratio and actual sex status.

Independent variables in total subjects	ASAA outcome		sIL-2R*α* outcome		sTNF-R1 outcome	
	(*N* = 142)		(*N* = 159)		(*N* = 159)	
	Beta^*∗*^	*p* values	Beta^*∗*^	*p* values	Beta^*∗*^	*p* values
17-OH P5	−0.039	0.725	0.021	0.863	0.088	0.327
DHEA	−0.113	0.519	0.093	0.463	0.228	0.015
Adione	0.080	0.490	−0.091	0.385	−0.241	0.002
DHEAS	0.141	0.323	0.012	0.928	−0.166	0.084
LH	0.092	0.312	0.016	0.887	0.052	0.528
E2/T ratio	0.270	<0.001	−0.040	0.694	−0.029	0.696
Sex (F = 0, M = 1)	−0.434	0.044	−0.333	0.075	0.682	<0.001
Entry age	0.194	0.032	−0.043	0.672	−0.066	0.371

Independent variables in control subjects	ASAA outcome		sIL-2R*α* outcome		sTNF-R1 outcome	
	(*n* = 113)		(*n* = 126)		(*n* = 126)	
	Beta^*∗*^	*p* values	Beta^*∗*^	*p* values	Beta^*∗*^	*p* values

17-OH P5	−0.113	0.377	0.101	0.456	0.085	0.380
DHEA	−0.094	0.631	0.143	0.296	0.230	0.024
Adione	0.173	0.174	−0.110	0.356	−0.257	0.004
DHEAS	0.089	0.622	−0.102	0.519	−0.110	0.341
LH	0.099	0.339	0.063	0.622	0.085	0.361
E2/T ratio	0.296	0.002	−0.074	0.515	0.006	0.942
Sex (F = 0, M = 1)	−0.321	0.215	−0.229	0.278	0.692	<0.001
Entry age	0.164	0.100	0.004	0.970	−0.053	0.537

Independent variables in pre-RA subjects	ASAA outcome		sIL-2R*α* outcome		sTNF-R1 outcome	
	(*n* = 29)		(*n* = 33)		(*n* = 33)	
	Beta^*∗*^	*p* values	Beta^*∗*^	*p* values	Beta^*∗*^	*p* values

17-OH P5	0.296	0.269	−0.105	0.762	−0.078	0.786
DHEA	0.071	0.892	0.053	0.893	0.136	0.671
Adione	−0.589	0.115	−0.096	0.729	−0.131	0.545
DHEAS	0.015	0.962	0.306	0.241	−0.283	0.193
LH	0.049	0.835	−0.295	0.240	−0.111	0.584
E2/T ratio	0.018	0.940	0.316	0.326	−0.476	0.073
Sex (F = 0, M = 1)	−0.734	0.215	−0.584	0.224	0.399	0.295
Entry age	0.198	0.432	−0.145	0.519	−0.118	0.506

^*∗*^Standardized beta coefficients from linear regression of log-transformed, reported NEI biomarkers.

**Table 7:** Linear regression of standardized immunologic and steroidal variables.

Sex of subjects and independent variables in pairs	Subjects in models^†^	Dependent variables in pairs of linear regression models^*∗*^									
		IL-1 beta		IL-1ra		Adione		Test		Cortisol	
		Beta	*p*	Beta	*p*	Beta	*p*	Beta	*p*	Beta	*p*
*Female subjects*											
Age	Pre-RA	−0.390	0.037	0.349	0.115	−0.538	0.002	0.054	0.799	0.027	0.913
	CN	−0.182	0.028	0.052	0.528	−0.350	<0.001	−0.002	0.983	0.160	0.107
IL-1 beta	Pre-RA	—	—	0.282	0.066	−0.004	0.984	0.425	0.029	0.221	0.341
	CN	—	—	0.539	<0.001	0.004	0.959	−0.104	0.204	0.266	0.009
IL-1ra	Pre-RA	0.385	0.066	—	—	0.233	0.126	−0.291	0.085	−0.069	0.730
	CN	0.531	<0.001	—	—	0.152	0.034	0.047	0.573	−0.243	0.017
Androstenedione	Pre-RA	−0.004	0.984	0.327	0.126	—	—	0.203	0.212	0.091	0.524
	CN	0.005	0.959	0.213	0.034	—	—	0.485	<0.001	0.092	0.122
Testosterone	Pre-RA	0.350	0.029	−0.328	0.085	0.253	0.212	—	—	−0.121	0.446
	CN	−0.112	0.204	0.049	0.573	0.638	<0.001	—	—	−0.031	0.650
Cortisol	Pre-RA	0.137	0.341	−0.058	0.730	0.151	0.524	−0.161	0.446	—	—
	CN	0.184	0.009	−0.166	0.017	0.188	0.122	−0.048	0.650	—	—
Variances explained^‡^	Pre-RA	0.420		0.208		0.436		0.296		0.063	
	CN	0.359		0.369		0.549		0.406		0.075	

*Male subjects*											
Age	Pre-RA	0.254	0.295	−0.355	0.173	−0.386	0.063	0.027	0.913	0.132	0.623
	CN	−0.130	0.303	−0.127	0.319	−0.177	0.058	0.160	0.107	0.128	0.193
IL-1 beta	Pre-RA	—	—	−0.162	0.545	0.381	0.131	−0.046	0.895	0.320	0.306
	CN	—	—	−0.023	0.854	0.045	0.630	−0.122	0.304	−0.055	0.572
IL-1ra	Pre-RA	−0.193	0.545	—	—	−0.337	0.145	−0.243	0.436	0.309	0.279
	CN	−0.023	0.854	—	—	−0.013	0.886	0.098	0.405	−0.018	0.853
Androstenedione	Pre-RA	0.472	0.131	−0.498	0.145	—	—	0.089	0.694	0.202	0.401
	CN	0.080	0.630	−0.024	0.886	—	—	0.199	0.035	0.599	<0.001
Testosterone	Pre-RA	−0.033	0.895	−0.212	0.436	0.150	0.694	—	—	0.428	0.160
	CN	−0.131	0.304	0.107	0.405	0.329	0.035	—	—	−0.051	0.736
Cortisol	Pre-RA	0.271	0.306	0.313	0.279	0.294	0.401	0.369	0.160	—	—
	CN	−0.089	0.572	−0.029	0.853	0.656	<0.001	−0.034	0.736	—	—
Variances explained^‡^	Pre-RA	0.535		0.445		0.624		0.363		0.451	
	CN	0.041		0.027		0.462		0.112		0.411	

^*∗*^Bivariate correlations of paired variables sequentially encountered in the first review of linear regressions were entered in the upper panel and the alternative correlations of the respective pairs were entered in the lower panel; all 10 paired correlational *p* values are equal in the upper and lower panels.

^†^Model sample sizes are female pre-RA = 36; CN = 144; male pre-RA = 18; and CN = 72.

^‡^Variances (*R* square) explained for the column variables as dependent outcomes in the 20 sex and subject models.

**(a):** 

Variables in model (variance extracted = 72.7%)	Principal components extracted			
	1^*∗*^	2	3	4
	(AA steroids)	(Receptors)	(IL-1*β*/ra)	(Cortisol)
Age at entry	**−.556**	.198	.250	.190
dhea_silW_new	**.870**	−.006	−.010	.209
adione_slW_new	**.871**	.048	−.050	.225
test_silW_new	**.767**	.079	.162	−.137
il_2_alplW_new	−.026	**.822**	.073	.107
stnf_r1lW_new	.042	**.865**	−.157	−.118
il_1_betlW_new	−.109	−.060	**−.881**	.185
il_1ralW_new	.131	.183	**−.722**	−.201
cort_silW_new	.115	.003	−.043	**.934**

Rotation method: Oblimin with Kaiser normalization, in 5 iterations.

^*∗*^Androgenic anabolic (AA) steroids are first extracted and inverse with age.

**(b):** 

Variables in model (variance extracted = 62.3%)	Principal components extracted		
	1^*∗*^	2	3
	(AA steroids)	(Receptors)	(Cortisol)
Age at entry	**−0.596**	−0.266	0.128
dhea_silW_new	**0.809**	−0.250	0.188
adione_slW_new	**0.851**	−0.137	0.020
test_silW_new	**0.623**	0.025	−0.380
il_2_alplW_new	−0.108	**0.816**	−0.043
stnf_r1lW_new	0.082	**0.822**	0.279
il_1_betlW_new	**0.688**	0.209	−0.065
il_1ralW_new	0.006	0.205	**0.905**
cort_silW_new	**0.504**	−0.150	0.213

Rotation method: Oblimin with Kaiser normalization, in 6 iterations.

^*∗*^IL-1*β* and cortisol are first extracted with the AA steroids.
